# Water Buffalo Responsiveness during Milking: Implications for Production Outputs, Reproduction Fitness, and Animal Welfare

**DOI:** 10.3390/ani12223115

**Published:** 2022-11-11

**Authors:** Madalina Mincu, Dinu Gavojdian, Ioana Nicolae, Alexandru Corneliu Olteanu, Adrian Bota, Constantin Vlagioiu

**Affiliations:** 1 Research and Development Institute for Bovine, Academy of Agricultural and Forestry Sciences, 077015 Balotesti, Romania; 2 Faculty of Veterinary Medicine, University of Agronomic Sciences and Veterinary Medicine of Bucharest, 011464 Bucharest, Romania; 3 Department of Computer Science and Engineering, University Politehnica of Bucharest, 060042 Bucharest, Romania; 4 Research and Development Station for Buffalo, Academy of Agricultural and Forestry Sciences, 507195 Sercaia, Romania

**Keywords:** water buffalo, behavior, milking temperament, animal welfare, production efficiency, infrared thermography, reproduction outputs

## Abstract

**Simple Summary:**

Human–animal interactions in dairy species are frequent, as milking is routinely performed twice per day. When buffalo cows are stressed, even by minor changes in their milking routine, a decline in the oxytocin supply is observed, which is strongly associated with reduced milk ejection. As a consequence, a high number of farms are administrating oxytocin injections to ensure complete emptying of the udder, which in turn impairs the resumption of a new ovarian cycle and represents an animal welfare concern. The overall objective of our study was to evaluate the effects of the milking temperament of water buffaloes on milking traits, animal-based welfare indicators, and reproductive performance. We found that milking temperament of buffalo cows has a significant influence on the milk production and on milking speed, with calmer animals outperforming their nervous counterparts. No effects of the milking temperament on reproduction efficiency and animal-based welfare parameters were observed. In conclusion, selection for calmer water buffalo would result in higher milk yields and improved milk ejection, while reducing the need of oxytocin administration, which would in turn improve reproduction and animal welfare.

**Abstract:**

The overall objective of this study was to evaluate the effects that milking temperament (MT) of water buffaloes has on milking traits, welfare indicators, and reproductive outputs. The study was performed on 60 multiparous buffalo cows (6.7 ± 0.6 lactations) at the beginning of their lactation (100 days in milk, DIM). Each buffalo cow was scored by two independent observers using a temperament scoring system (1: extremely calm, 2: calm, 3: alert, 4: reactive, and 5: aggressive), and then grouped as ‘calm’ (scores 1, 2, and 3; *n* = 42) or ‘nervous’ (scores 4 and 5; *n* = 18). Additionally, the milk yield at 100 DIM (MY), milking speed (MS), calving interval (CI), age at first calving (AFC), body condition score (BCS), animal-based welfare parameters, and infrared thermography data (IRT) were evaluated. The MT significantly influenced the MY (*p* = 0.0082), with calmer cows outperforming their nervous counterparts. The MS was significantly influenced by the MT (*p* = 0.0015), with calmer animals having a higher milk ejection rate. The MT of the cows had no influence on the CI, AFC, or BCS. The evidence from this study suggests that the responsiveness of buffalo cows during milking affects their milk yield and milking speed, with no associations being found for reproduction efficiency indicators or animal-based welfare indicators.

## 1. Introduction

In highly gregarious farm animals such as cattle and water buffalo, the social environment is an important determinant of their welfare and overall health fitness [1]. It was shown that some large domestic ruminants are able to make sophisticated discriminations between conspecifics and humans, possess emotional contagion, and have distinct personalities, while exhibiting dimensions of social complexity, such as social learning [2,3,4]. 

Human–animal interactions in dairy species are frequent, as milking is routinely performed more than once per day, while being strongly correlated with the animals’ performance and welfare status [5]. Behavioral reactivity (temperament) is commonly assessed by visual scores that consider the frequency and intensity of animal movements, which subsequently reflect fear levels and responsiveness [6]. Lactating water buffalo cows are known to be more sensitive to handling during milking than dairy cows, due to the fact that they were less intensively selected for milking traits [7,8]. Previous studies highlighted that pre-milking stimulation and avoidance of stress during milking are prerequisites for the alveolar milk fraction ejection in buffalo cows, which represents 90–95% of their entire milk production [9]. Moreover, temperamental traits have economic implications in farmed dairy species [10]. 

When buffalo cows are stressed, even by minor changes in their milking routine, a secretion of adrenaline is induced, leading to a decrease in the oxytocin supply, which is strongly associated with a reduced milk ejection in the species [11]. The milking temperament of dairy buffalo cows has been found to significantly influence the milk yield, fat yield, and somatic cell count [12,13]. Furthermore, negative human–animal interactions during milking were shown to be strongly correlated with restless stepping and kicking in buffalo cows [14].

Given that breeding and selection have failed to increase the milk ejection in buffaloes, most large farms use oxytocin injections to improve the milk let-down reflex [14,15]. A previous study demonstrated that repeated injections of oxytocin interfere negatively with the normal milk secretory activity of the mammary epithelium, while inhibiting the normal ejection reflex [16]. Furthermore, it was highlighted that exogenous oxytocin administration has a negative effect on the onset of a new ovarian cycle in buffalo cows, causing numerous fertility disorders, such as poor estrus signs, low conception rates, reduced lactation length, and high embryonic mortality [17]. On the other hand, under small-scale farming, it is common for the milking to be performed manually and in the presence of the calf, given the strong dam–calf pair bonding and the imprinting mechanisms found in water buffalo [18].

To date, research on the implications of temperament on production, reproduction, and welfare in dairy water buffalo is scarce, in contrast to the strong body of literature available for dairy and beef cattle, where temperament was included as a selection trait into breeding schemes for a number of breeds [19,20].

The overall objective of this study was to evaluate the effects that milking temperament (MT) of dairy water buffalo has on milking traits, animal-based welfare indicators, and reproductive efficiency. Our hypothesis was that calmer and less excitable buffalo cows would outperform their more reactive and nervous counterparts, when taking into account the production and reproduction outputs.

## 2. Materials and Methods

### 2.1. Animal Management and Temperament Assessment

The study was carried out at the Research and Development Station for Buffaloes, Sercaia (GPS: 45°50′ N 25°8′ E), Romania (altitude of site 445 m), on 60 multiparous dairy buffalo cows (6.7 ± 0.6 lactations; Romanian buffalo breed, *Bivolul Românesc* national name) at the beginning of their lactation (100 days in milk, DIM), between May and August 2022.

The animals were managed under identical feeding and housing conditions; so far, no selection for temperament of the buffalo cows has been practiced at the RD station. A dataset with 17 parameters per buffalo cow was set up and further analyzed for evaluating the effects of MT on milk yield and milking speed, reproduction, and animal-based welfare indicators. As nighttime, the cows were housed in a tie-stall barn, using wheat straws as bedding, while having ad libitum access to water and mineral blocks. In the daytime, the animals had access to a natural pasture for 12 h/day, with no supplementation of their ration with hay or concentrates during the summer season. The buffalo cows were milked twice per day inside the tie-stall barn, starting at 5:00 a.m. and 6:00 p.m., using individual milking machines, with separation from calves occurring at 7 days postpartum. The milking protocol was as follows: wiping off the teats and udder massage, fore-stripping into a cup, attachment of the milking unit (1–2 min between beginning of the milking process and the attachment of the machine), monitoring the milk flow, massaging the udder for reducing the residual milk, manually reattaching the milking machine kicked off by cows, stopping the vacuum flow when milking was complete, detaching the milking unit, and post-dipping of the teats. The research station did not practice oxytocin administration pre-milking in the last 2 years, given the negative effects on postpartum reproduction outputs of such a practice, with low-stress and gentle handling methods being always implemented by the stockperson during milking.

MT of the animals was assessed adapting the method described by [21] for dairy cows, using two individual trained observers placed at 1–1.5 m behind the animals during milking. Consensus was reached at the end of each milking session, while the final temperament score for each animal was given by comparing individual observations and notes. Scoring of MT was performed only once per cow during the first 40 days of lactation.

MT of animals was evaluated using a five-point score, as follows:

1. The buffalo cow is ruminating, relaxed and extremely calm, no movement;
2. The buffalo cow is alert but calm, with occasional head and ear movements;
3. The buffalo cow is alert and reactive to the milking machine being put on and taken down, with moderately movements of hind legs;
4. The buffalo cow kicks and pendulates her gate from one hind leg to another, defecates, and/or urinates, with abrupt episodic movements;
5. The buffalo cow kicks and tries to take the milking machine down, is obviously restless, emits vocalizations, and defecates/urinates, with permanent episodic movements, head butting aggressively.

Buffalo cows were classified on the basis of their temperament as either ‘calm’ (*n* = 42; scores 1, 2, and 3), or ‘nervous’ (*n* = 18; scores 4 and 5). 

All procedures were approved by the Research and Development Institute for Bovine Institutional Review Board (approval code PN-III-P1-1.1-TE-2021-0027), with the behavioral temperament assessment and subsequent recordings causing low distress to the buffalo cows, given the presence during milking sessions of the two unfamiliar observers.

### 2.2. Data Collection and Statistical Analysis

Ear-tag number, milk yield per milking session (kg), and milk duration (min) were collected directly during the temperament assessment. Milking speed (kg/min) for each animal was obtained by dividing the milk yield to milking duration.

Infrared thermography (IRT) data were taken pre- and post-milking during the behavioral assessment days, using an FLIR ONE Pro LT mobile camera (19,200-pixel resolution, temperature range −20 °C to 400 °C) and FLIR Systems INC^©^ image processing software. Temperature measuring points were the lacrimal caruncle of the eye in the orbital region (*regio orbitalis*) and at the nasal region (*regio nasalis*), which were previously validated as thermal windows for water buffalo [22], with IRT pictures being taken (twice per animal per region) from a 1.8 m distance, following the manufacturers recommendations.

The daily milk yield per water buffalo cow (kg/day) was measured twice during the period of data collection, according to the standardized International Committee for Animal Recording guidelines for dairy buffaloes [23].

Body condition score (BCS) was assessed using a nine-point scale, where 1 was severely emaciated, no presence of fat either visible or palpable, and physically weak and 9 was severely obese with typical ‘fat pads’, in increments of 1 according to a previously developed scale [24]. Given that BCS assessment was performed using a nine-point parametric scale, we classified the animals as follows: buffaloes with thin/low BCSs (scores 1, 2, and 3), buffaloes with average BCSs (scores 4, 5, and 6), and buffaloes with fat/high BCS (scores 7, 8, and 9).

Cleanliness of the udder, rump, and hind legs (scores 0—no dirt or minor splashing, 1—intermediate, or 2—separate or continuous plaques of dirt) was evaluated for each individual buffalo cow according to the Welfare Quality^®^ protocol for dairy cattle [25]. Tarsal joint and skin lesion incidence was evaluated during the behavioral assessment of the animals. Claw overgrowth as a lameness indicator was evaluated as follows: 0—mild growth, 1—medium growth, or 2—severe growth, using a method previously described [26]. Additionally, integument alterations such as hairless patches (scores: 0—no hairless patch, 1—at least one hairless patch, or 2—more than one hairless patch), and nasal, ocular, and vulvar discharges were assessed at an individual level: 0—no evidence of nasal/ocular/vulvar discharge or 2—evidence of nasal/ocular/vulvar discharge. 

Reproductive outputs of the buffalo cows (age at first calving and calving intervals) were recorded by the research station veterinarians and technicians. 

Comparisons between the two temperament classes (calm and nervous) for milk yield, milking speed, calving interval, age at first calving, and pre- and post-milking IRT were carried out using the nonparametric Mann-Whitney U test, given that the Shapiro-Wilk tests showed a significant departure from normality.

The chi-square test of independence was performed to determine if temperament had an influence on the BCS, cleanliness of udder, cleanliness of rump, cleanliness of hind legs, claw overgrowth, hairless patches, tarsal joint lesions, skin lesions, and nasal, ocular, or vulvar discharges.

The correlation between milk yield and milking speed with respect to the temperament classes was explored using linear regression models. The two-way analysis of variance (ANOVA) was used to study how milk yield, calving interval, and age at first calving are influenced by BCS and MT as independent variables. Inconsistencies in the final database (missing or abnormal data) led to the exclusion of three animals when calculating the calving interval, and of one animal for the age at first calving. 

All statistical inferences were carried out using Minitab17 software (Minitab LLC^®^) and Microsoft Excel. Decisions about the acceptance or rejection of the statistical hypothesis were made at the 0.05 level of significance.

## 3. Results

MT of the buffalo cows significantly influenced the milk yield (*p*-value = 0.0082), with the calmer cows having a higher milk yield than their nervous counterparts. The difference between the two classes of temperament regarding the milk yield was an average of 331.3 kg milk/100 DIM, as shown in Table 1. The milking speed was significantly influenced by the MT of buffalo cows (*p*-value = 0.0015), with calmer animals having a faster milk ejection rate when compared to more reactive animals. Calving interval (CI) was not influenced by MT (*p* > 0.05) in our study; nevertheless, CI was 14.8 days longer in calm cows. Buffalo cows age at first calving was not influenced by the temperament (*p* > 0.05); however, this trait tended to extend from 51.12 months in calm buffaloes to 56.5 months in nervous buffaloes.

**Figure 1:**
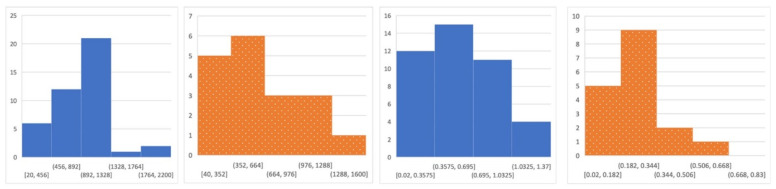
Histograms of milk yield (**left**) and milking speed (**right**) for calm (blue) and nervous (orange) buffalo cows.

The Shapiro–Wilk tests showed a significant departure from normality (for milk yield in calmer cows W(42) = 0.94, *p* = 0.029, for milking speed in calmer cows W(42) = 0.929, *p* = 0.012 and in nervous cows W(18) = 0.867, *p* = 0.016), proving the effectiveness of the Mann-Whitney U-test applied. Figure 1 shows that both calm and nervous groups exhibit right-skewed distributions in terms of milk yield and milking speed.

The BCS was not influenced by the MT class in our study (chi-square test, *p* > 0.05), leading us to treat MT and BCS as factors using a two-way ANOVA approach. When considering the BCS percentages among classes of temperament, a tendency for thin BCS in nervous animals was observed (Table 2).

No differences were found between calm and nervous animals regarding the percentage of ocular discharge (*p* > 0.05) or nasal discharge (*p* > 0.05). Likewise, vulvar discharge incidence was not influenced by the behavioral reactivity of the animals in our study; worth mentioning is that, in nervous buffalo cows, the percentage of vulvar discharge appearance was null and the usage of a chi-square test would have not been appropriate. Considering the percentage of the buffalo skin lesions, no difference among temperament groups was observed in our study (*p* > 0.05).

No significant differences were observed between calm and nervous buffalo cows, when taking into consideration the percentage of tarsal joint lesions (*p* > 0.05). Likewise, the analysis did not reveal any significant differences between temperament classes in terms of hairless patches (*p* > 0.05); data are presented in Table 3. Similarly, no differences were observed in our study based on MT with regard to cleanliness of the rump, udder, or hind legs. MT of the buffalo cows had no influence on the percentage of the overgrown claws; worth mentioning is that the usage of the chi-square test would have not been appropriate on our data, as there were no calm buffalo cows with excessively overgrown claws.

**Figure 2:**
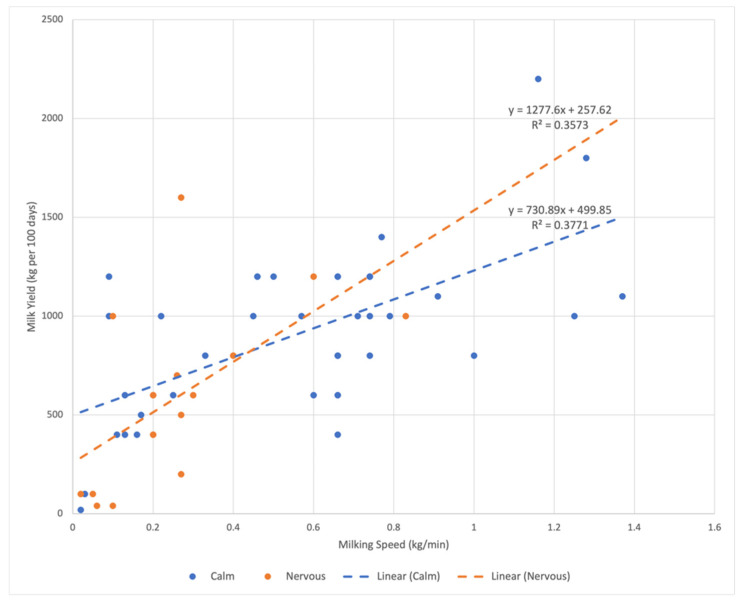
Linear regression models for the dependency between milk yield and milking speed.

We further explored the effects of MT on the correlation between milk yield and milking speed. First, we computed the Pearson correlation coefficient between milk yield and milking speed in the cohort and found a strong positive correlation between the two traits (r = 0.652). A linear regression was then used to model the relationship between milk yield and milking speed for calm buffalo cows (y = 730.89x + 499.85, R^2^ = 0.3771) and nervous buffalo cows (y = 1277.6x + 257.62, R^2^ = 0.3573), which showed that, for nervous cows, the dependency of milk yield on milking speed was considerably steeper (Figure 2), with lower milking speed corresponding to lower milk yields for nervous cows. In our dataset, the maximum milking speed for nervous cows (0.83 kg/min) was considerably lower than that for calmer cows (1.37 kg/min), which explains the significantly higher milking speed for calmer buffalo cows. 

Furthermore, we used the two-way analysis of variance (two-way ANOVA) to study how milk yield, calving interval, and age at first calving are influenced by BCS and MT, which we considered to be independent variables, since the chi-square test failed to show any dependency. Table 4 shows the results in testing the dependency of milk yield, calving interval, and age at first calving on (A) MT and (B) BCS. We found a significant dependency of milk yield toward temperament (F = 7.5985, *p* = 0.008), which also confirms our results from the Mann-Whitney U-test, and no dependency toward BCS. The results in testing the dependency of calving interval on (A) MT and (B) BCS illustrates that the dependency toward temperament cannot be proven significant (*p* = 0.8637); however, the dependency toward BCS (F = 2.4897, *p* = 0.093) and toward both MT and BCS (F = 2.5329, *p* = 0.0894) had a tendency toward significance, if we consider a threshold at *p* ≤ 0.10. Thus, we found that calmer buffalo cows with higher BCSs have an average of 830 days for the calving interval, significantly higher than the other groups, ranging between 396 and 442 days. Lastly, the results of testing the dependency of age at first calving on (A) MT and (B) BCS showed that the dependency toward temperament could not be proven significantly, neither could the dependency toward temperament and BCS (*p* = 0.7193); however, the dependency toward BCS (F = 3.3385, *p* = 0.0431) was significant (*p* ≤ 0.05).

Regarding the nasal and orbital IRT temperature pre- and post-milking, no statistical influence of the temperament class (*p* > 0.05) was observed between calm and nervous animals (Table 5). However, in calm buffalo cows, the nasal IRT temperature dropped on average by 0.15 °C post milking, while, for their nervous counterparts, the nasal IRT temperature was higher by 0.84 °C after milking, which could be attributed to higher stress levels faced by the excitable animals during milking. A similar pattern was observed for the orbital IRT temperature, when, for calm cows, the temperature post milking dropped on average by 0.15 °C, while, in nervous buffalo cows, the IRT temperature was higher by 0.32 °C. 

## 4. Discussion

The responsiveness of buffalo cows during milking and, subsequently, the effects of MT on milk yield during the first 100 days of lactation are consistent with previous reports [12,27], where docile buffalo cows had higher daily milk yields, when compared to their nervous counterparts. Furthermore, our data are in complete agreement with those of Bharadwaj et al. [28], on milk yield distribution among temperament classes in dairy buffalo, with the amendment that the authors included three temperament classes in their study, namely, docile, nervous, and aggressive buffalo cows. 

A potential explanation for the reduced milk yields of nervous buffalo cows could be attributed to the higher adrenalin secretion [12,29], which subsequently led to an incomplete milking, with higher quantities of residual milk stored in the alveolar fraction of the buffalo cow udder.

The milking speed was influenced by the behavioral reactivity of the buffalo cows in our study, with similar results being reported for the species [27,30]. In addition to the more obvious advantages of high milking speed in buffalo cows, such as the reduction in milking session duration or an increase in the number of cows milked per stockperson, delayed milk ejection affects the health of the udder by causing a vacuum in the milk flow before the cistern is empty, alongside blood flow interruptions, allowing air to enter in the mammary gland and, thus, increasing the exposure to bacteria at the end of the teats [31].

Moreover, it was hypothesized that reactive buffalo cows exhibit greater levels of stress when compared to dairy cattle, having a higher teat sensitivity to milking, given that they have only recently been introduced to machine milking and early calf separation [14]. 

The lack of significant relationships between MT and reproduction parameters contradicts our hypothesis, which was that an animal that is more reactive during milking would have inferior reproduction efficiency. While considering the relative short duration of the lactation in water buffalo, of 270 days for the standard lactation, both AFC and CI are of outmost importance for the dairy buffalo sector, since calving is conditioning the onset of a new lactation, with both parameters having significant economic implications. To date, no study has addressed the association between MT and the reproduction efficiency of water buffalo cows. Lack of differences for AFG and CI in the current study might be attributed to the masking effects of water buffalo herd hierarchy, with aggressive and nervous animals being more dominant, thus giving priority to feeding and resting areas, potentially resulting in higher feed intake and lower levels of stress, compared to dominated buffalo cows, with both nutrient availability and stress hormones being known to influence reproduction [32,33].

Contrary to our results on CI and AFC duration, previously published articles reported shorter intervals for both parameters [34,35,36]; however, it is worth mentioning that they evaluated reproduction of water buffalo under different climatic regions, significantly warmer than those found in our study, with the Romanian buffalo being mainly reared under highland conditions of the Carpathian mountains, which is expected to negatively influence both age at sexual maturity of heifers and resumption of a new estrous cycle after calving.

Regarding the infrared thermography data (IRT), although we found no statistical differences between nasal and orbital pre- and post-milking temperatures, our results on temperature changes in both thermal windows are in accordance with those previously reported in cattle when faced to thermal stress by exposing the animals to direct solar radiation [37], and in line with the reports of Mota-Rojas et al. [22] on IRT temperature changes and current limitations in water buffalo stress evaluation. The relatively low number of animals available for this study might have contributed to these inconsistent results and to the lower statistical sensitivity.

In the current study, we found no relationship between MT of buffalo cows and animal-based welfare indicators. However, animal behavior and human–animal interactions are themselves important indicators when evaluating welfare at the farm level. With the improvement of animal wellbeing in modern animal husbandry becoming a major societal issue and a priority for both research and practice, it is necessary to develop reliable science-based tools that allow direct assessment at animal level, in order to assess the situation on farm or to test alternative farming conditions that improve welfare. Such new approaches could be represented by the study and analysis of vocal parameters emitted by the buffalo cows in anticipation and during milking. Unlike in dairy cattle, we observed a high incidence of vocalizations emitted during the milking sessions of the studied buffalo cows, with animals using both high- and low-frequency calls. Vocal parameters are recognized as feasible indicators for stress and welfare assessment in farmed animals such as pigs, laying hens, and horses [38], with a substantial lack of knowledge in water buffalo communication behavior.

There were some limitations to our study, since it cannot be ruled out that there was some unintended bias in our trial, considering that the animals were milked by two animal caretakers, as well as the strong bonds that buffalo cows build with their stockpersons, and that the animal-human relationship plays a significant role in behavior expressed during milking [11,14,33]. Moreover, given the different selection programs from various countries in which water buffalo are being reared, following the findings from cattle [10], differences among breeds in terms of MT and aggressiveness levels are to be expected.

On the basis of the current findings, it can be emphasized that further studies are needed to predict the heritability of MT and the genetic correlation between production and reproductive performance and animal behavior.

## 5. Conclusions

We conclude that the milking temperament of buffalo cows has strong effects on the overall milkability of the species, specifically on milk yield during the first 100 days of lactation and on milking speed. Although reactiveness during milking has not been associated with the main reproduction efficiency parameters or the animal-based welfare indicators, both of these group traits can be used in an integrated manner for a broader assessment of the temperament of these animals and its implications. 

Our study provides a blueprint for including milking responsiveness of dairy water buffalo as an independent selection trait in future genetic improvement programs, considering both the economic implications and the feasibility of assessing milking temperament at farm level, with no additional costs during performance recordings or infrastructure needed.

The importance of our work lies in the improvement of both milk yields and milking speed in the species, while also having the potential to improve animal welfare, by selecting animals which are less fearful, thereby exhibiting lower levels of stress during the entire production life and, thus, coping better with the environment.

These findings add to a growing body of literature on the effects of farmed animal behavior on production, reproduction, and welfare, which in the future can assist the development of new management practices integrating the behavioral processes and needs of dairy water buffalo.

## Figures and Tables

**Table 1:** Mean ± SEM for milk yield, milking speed, calving interval, and age at first calving in calm and nervous buffalo cows.

Table 100.	Milk Yield (kg/100 DIM)	Milking Speed (kg/min)	Calving Interval (days)	Age at First Calving (months)
Cohort	828.8 ± 57.8	0.48 ± 0.044	499.2 ± 41.8	52.76 ± 2.75
Calm	924.3 ± 63.6 ^a^	0.58 ± 0.053 ^a^	503.9 ± 50.5	51.12 ± 2.96
Nervous	593.0 ± 108.0 ^b^	0.26 ± 0.047 ^b^	489.1 ± 76.5	56.50 ± 6.04
Significance	*p* = 0.0082	*p* = 0.0015	*p* = 0.8962	*p* = 0.6092

SEM, standard error of the mean; DIM, days in milk. Column means with different superscript differ significantly at *p* ≤ 0.05.

**Table 2:** Contingency tables for body condition score, ocular discharges, skin lesions, and nasal discharges in calm and nervous buffalo cows.

Temperament	Body Condition Score (BCS) %			Ocular Discharges %		Skin Lesions %		Nasal Discharges %	
Welfare Quality^®^ scale	Thin (1–3)	Average (4–6)	Fat (7–9)	0	1	0	1	0	1
Cohort	41.66	35.00	23.33	91.66	8.33	95.00	5.00	95.00	5.00
Calm	33.33	38.09	28.57	92.85	7.14	97.61	2.38	95.23	4.76
Nervous	61.11	27.77	11.11	88.88	11.11	88.88	11.11	94.44	5.55
Significance	NS, *p* = 0.1128			NS, *p* = 0.6102		NS, *p* = 0.1550		NS, *p* = 0.8971	

NS—not significant.

**Table 3:** Contingency tables for tarsal joint lesions, hairless patches, cleanliness of rump, cleanliness of udder, and cleanliness of hind legs in calm and nervous buffalo cows.

Temperament	Tarsal Joint Lesions %			Hairless Patches %		Cleanliness of					
						Rump %		Udder %		Hind Legs %	
Welfare Quality^®^	0	1	2	0	1	0	2	0	2	0	2
Cohort	90.00	5.00	5.00	31.66	68.33	71.66	28.33	88.33	11.66	45.00	55.00
Calm	92.85	4.76	2.38	30.95	69.04	66.66	33.33	90.47	9.52	45.23	54.76
Nervous	83.33	5.55	11.11	33.33	66.66	83.33	16.66	83.33	16.66	44.44	55.55
Significance	NS, *p* = 0.2597			NS, *p* = 0.8558		NS, *p* = 0.1892		NS, *p* = 0.4296		NS, *p* = 0.9548	

**Table 4:** Results of two-way ANOVA in testing the dependency of milk yield, calving interval, and age at first calving on (A) milking temperament and (B) body condition score.

	Source of Variation	Degrees of Freedom DF	Mean Squares MS	F	*p*-Value
Milk yield	A	a − 1 = 1	1,380,071.4286	7.5985	0.0080
	B	b − 1 = 2	26,968.6667	0.1485	0.8624
	AB	(a − 1) (b − 1) = 2	122,175.4892	0.6727	0.5146
	Error (residual)	n − ab = 54	181,624.8196		
Calving interval	A	a − 1 = 1	2717.6284	0.0298	0.8637
	B	b − 1 = 2	227,151.5682	2.4897	0.0930
	AB	(a − 1) (b − 1) = 2	231,094,2865	2.5329	0.0894
	Error (residual)	n − ab = 51	91,237.5818		
Age at first calving	A	a − 1 = 1	361.7877	0.8547	0.3594
	B	b − 1 = 2	1413.2408	3.3385	0.0431
	AB	(a − 1) (b − 1) = 2	140.3463	0.3315	0.7193
	Error (residual)	n − ab = 53	423.3154		

**Table 5:** Mean ± SEM for pre- and post-milking infrared thermography (IRT) data at orbital and nasal regions, in nervous and calm buffalo cows.

**Temperament**	**Nasal IRT Temperature (°C)**		**Orbital IRT Temperature (°C)**		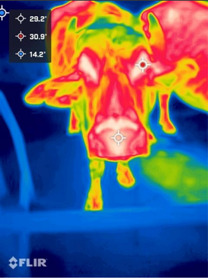
	**Pre-Milking**	**Post-Milking**	**Pre-Milking**	**Post-Milking**	
Cohort	29.33 ± 0.296	29.47 ± 0.392	31.75 ± 0.192	31.74 ± 0.422	
Calm	29.46 ± 0.305	29.31 ± 0.532	31.76 ± 0.263	31.61 ± 0.591	
Nervous	29.02 ± 0.734	29.86 ± 0.420	31.74 ± 0.209	32.06 ± 0.289	
Significance	NS, *p* = 0.916	NS, *p* = 0.712	NS, *p* = 0.958	NS, *p* = 0.958	

## Data Availability

The data presented in this study are available on request from the corresponding author.
